# Oriented Immobilization of His-Tagged Protein on a Redox Active Thiol Derivative of DPTA-Cu(II) Layer Deposited on a Gold Electrode—The Base of Electrochemical Biosensors

**DOI:** 10.3390/s130911586

**Published:** 2013-09-03

**Authors:** Edyta Mikuła, Magdalena Sulima, Ilona Marszałek, Aleksandra Wysłouch-Cieszyńska, Peter Verwilst, Wim Dehaen, Jerzy Radecki, Hanna Radecka

**Affiliations:** 1 Institute of Animal Reproduction and Food Research of Polish Academy of Sciences, Tuwima 10, Olsztyn 10-748, Poland; E-Mails: e.mikula@pan.olsztyn.pl (E.M.); j.radecki@pan.olsztyn.pl (J.R.); 2 Institute of Biochemistry and Biophysics of Polish Academy of Sciences, Pawińskiego 5a, Warsaw 02-106, Poland; E-Mails: magdalena.sulima@o2.pl (M.S.); ilamarszalek@gmail.com (I.M.); olawyslouch@ibb.waw.pl (A.W.-C.); 3 Chemistry Department, University of Leuven, Celestijnenlaan 200F, Leuven B-3001, Belgium; E-Mails: p.verwilst@ism.u-bordeaux1.fr (P.V.); Wim.Dehaen@chem.kuleuven.be (W.D.)

**Keywords:** pentetic acid-Cu(II) complex, gold electrodes, His-tagged proteins, Aβ peptides, electrochemical biosensor

## Abstract

This paper concerns the development of an electrochemical biosensor for the determination of Aβ_16–23′_ and Aβ_1–40_ peptides. The His-tagged V and VC1 domains of Receptor for Advanced Glycation end Products (RAGE) immobilized on a gold electrode surface were used as analytically active molecules. The immobilization of His_6_–RAGE domains consists of: (i) formation of a mixed layer of *N*-acetylcysteamine (NAC) and the thiol derivative of pentetic acid (DPTA); (ii) complexation of Cu(II) by DPTA; (iii) oriented immobilization of His_6_–RAGE domains via coordination bonds between Cu(II) sites from DPTA–Cu(II) complex and imidazole nitrogen atoms of a histidine tag. Each modification step was controlled by cyclic voltammetry (CV), Osteryoung square-wave voltammetry (OSWV), and atomic force microscopy (AFM). The applicability of the proposed biosensor was tested in the presence of human plasma, which had no influence on its performance. The detection limits for Aβ_1–40_ determination were 1.06 nM and 0.80 nM, in the presence of buffer and human plasma, respectively. These values reach the concentration level of Aβ_1–40_ which is relevant for determination of its soluble form in human plasma, as well as in brain. This indicates the promising future application of biosensor presented for early diagnosis of neurodegenerative diseases.

## 1. Introduction

Alzheimer's disease (AD) is the most common form of dementia resulting in memory deficits and confusion. The spread of AD doubles every five years after the age of 60, with estimates being over 20% in those over 80 years [1,2]. The amyloid hypothesis postulating the fundamental role of Aβ peptides in the development of the disease is mostly accepted [3–7]. Therefore, the Aβ peptide could be a potential biomarker of AD development [1,8].

The most frequently used techniques for determination of the concentration of the Aβ peptide at its early stage of aggregation are surface plasmon resonance (SPR) [9], nanoscale liquid chromatography [10], capillary electrophoresis [10], surface-enhanced Raman spectroscopy (SERS) [3], and electron microscopy [11,12]. All of them are very sensitive and selective, but rather expensive and time consuming, making these techniques usually unavailable in most medical laboratories.

The main objective of the research presented in this article was the development of an electrochemical biosensor for the detection of Aβ peptides based on a redox active layer consisting of a DPTA-Cu(II) complex. The DPTA–Cu(II) complex plays the role of molecular connector between the gold electrode surface and immobilized His_6_–RAGE V and His_6_–RAGE VC1 domains as well as transducer of the intermolecular recognition process. The crucial parameter deciding the sensitivity and selectivity of biosensors based on a redox active layer containing the proteins applied as the recognition elements is their oriented immobilization on the electrode surface. The immobilization procedure must maintain the molecule responsible for biorecognition close to the transducer surface, while retaining its biological activity in a reproducible manner. The immobilization layer should give the biological molecule an enhanced stability. It should be robust providing the control of distribution and orientation of the immobilized species [13–20].

Taking into account the above parameters, as a molecular connector for immobilization of His_6_–RAGE domains on the surface of electrode, the DPTA–Cu(II) complex was chosen. The DPTA–Cu(II) complex binds to histidine with an average binding constant of 4.5 × 10^3^ M^−1^, which corresponds to a binding energy of 4.8 kcal/mol [21]. The imidazole moieties of the His–tag can chelates the free coordination sides of Cu(II) and form the charge–neutral, octahedral DPTA-Cu(II)–His–tag complexes [15,22,23].

Receptor for Advanced Glycation End Products (RAGE) belongs to the multiligand receptors and is involved in many pathological processes such as neurological disorders, amyloidoses, stroke, and diabetic complications [24,25]. RAGE consists of three extracellular immunoglobulin domains: V, C1, C2, a transmembrane helix and a short (42 amino acids) tail and is responsible for the active transport of the Aβ peptide across blood-brain barrier [26].

The proposed biosensor was applied for screening of the interactions between RAGE V domain and shortened terminally blocked fragment of Aβ_16–23′_ (_Ac_KLVFFAED_NH2_) [1]. Ac and NH_2_ denote blocking of peptide termini at N and C-terminal end, respectively. This fragment of Aβ peptide was selected because the major binding site of the RAGE V domain was localized at the 7 amino acid stretch of residues of this sequence. Nearly the same region of Aβ_16–23′_ has been found to be indispensable for peptide aggregation into amyloid [1]. The next analyte selected was the Aβ_1–40_ peptide as a potential marker of Alzheimer's disease. To confirm that the observed analytical signals generated by the described biosensors originate from the recognition process between the RAGE V and VC1 domains and the Aβ peptides, the electrode modified with the mutated His_6_–RAGE V and His_6_–RAGE VC1 domains which have no ability to bind Aβ peptides were applied. The applicability of proposed biosensor was finally tested in the presence of human plasma.

## 2. Experimental Section

### 2.1. General

NMR spectra were recorded on a Bruker Avance 300 instrument in CDCl_3_. Chemical shifts (*δ*) are reported relative to TMS and coupling constants (*J*) are given in Hertz (Hz). Low-resolution mass spectra were recorded on a Thermo Finnigan LCQ Advantage (ESI).

### 2.2. Materials and Chemicals

*N*-Acetylcysteamine (NAC), TRIS-hydrochloride, copper (II) acetate, chloroform, potassium chloride, sodium chloride were obtained from Sigma-Aldrich (Poznań, Poland). Methanol, potassium hydroxide, sulfuric acid were purchased from POCH (Gliwice, Poland). Aβ_1–40_ was overexpressed in bacteria as fusions and then purified with reversed phase high-pressure chromatography. Aβ_16–23′_ was obtained from chemical synthesis [1]. Domain V 23–143 (with His_6_-tag at C-terminus) and its mutant V 23–143 K43K44R104/A were overexpressed in *Escherichia coli* strain BL21(DE3)-RIL and purified with Ni-NTA agarose [1]. All aqueous solutions were prepared using MilliQ water, resistivity 18.2 MΩcm (Millipore, Billerica, MA, USA). All solutions were deoxygenated by purging with nitrogen (ultra-pure 6.0, Warsaw, Poland) for 15 min. All experiments were carried out at room temperature.

### 2.3. Synthesis of Pentetic Acid (DPTA) Thiol Ligand

The thiol derivative of pentetic acid (DPTA) was synthesized at the Chemistry Department of Leuven University, Belgium. Briefly, to a stirred solution of *N*,*N*-bis{*N*,*N*-bis[(*tert*-butoxycarbonyl)-methyl]-ethylamine}-glycine [27] (500 mg, 0.81 mmol) in DCM_dry_ (100 mL) was added S-trityl cysteamine [28] (387 mg, 1.21 mmol), HATU (342 mg, 0.90 mmol) and DIPEA (425 μL, 2.44 mmol) and the reaction mixture was subsequently stirred at room temperature overnight. The resulting mixture was washed with NaHCO_3,sat. aq._ (100 mL) and brine (100 mL). The organic layer was dried over Na_2_SO_4_ and column chromatography (silica gel, DCM/EtOAc) yielded 331 mg (0.36 mmol, 45% yield) of the protected ligand.


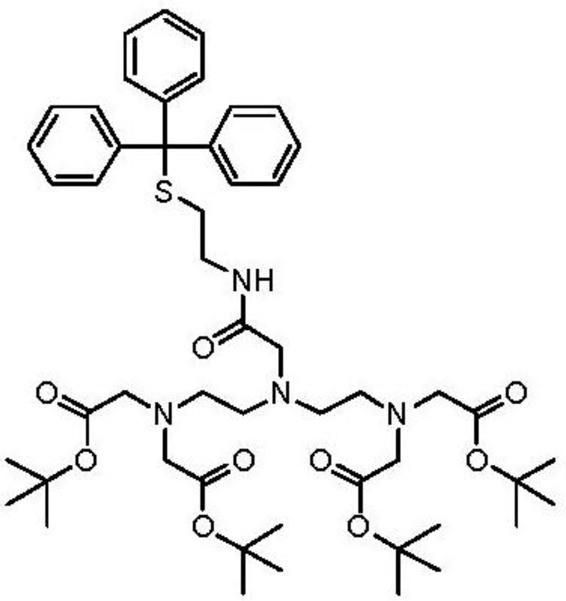


^1^H-NMR (CDCl_3_): *δ* 1.44 (s, 36H), 2.38 (t, *J* = 7.1 Hz, 2H), 2.58 (t, *J* = 6.2 Hz, 4H), 2.73 (t, *J* = 6.2 Hz, 4H), 3.06 (s, 2H), 3.15–3.24 (m, 2H), 3.32 (s, 8H), 7.15–7.32 (m, 9H), 7.41 (d, *J* = 7.6 Hz, 6H), 8.18 (br. t, *J* = 6.0 Hz, 1H) ppm. ^13^C-NMR (CDCl_3_): *δ* 28.19, 31.76, 38.15, 51.97, 53.63, 55.90, 58.47, 66.47, 81.01, 126.59, 127.87, 129.62, 144.91, 170.51, 171.74 ppm. LRMS (ESI): [M] = C_51_H_74_N_4_O_9_S, *m*/*z* 942 [M+Na]^+^, 920 [M+H]^+^.

To a solution of protected ligand (331 mg, 0.26 mmol) in DCM_dry_ (10 mL) and TFA (20 mL) was added triisopropylsilane (53 μL, 0.26 mmol), after which the solution was stirred overnight at room temperature. The solvent was removed *in vacuo* and the crude oil was partially dissolved in H_2_O (50 mL). The suspension was filtered and the solvent was removed *in vacuo* resulting in 37 mg (0.08 mmol, 31% yield) of the deprotected ligand which was used without further purification. LRMS (ESI): [M] = C_16_H_28_N_4_O_9_S, *m*/*z* 475 [M+Na]^+^.


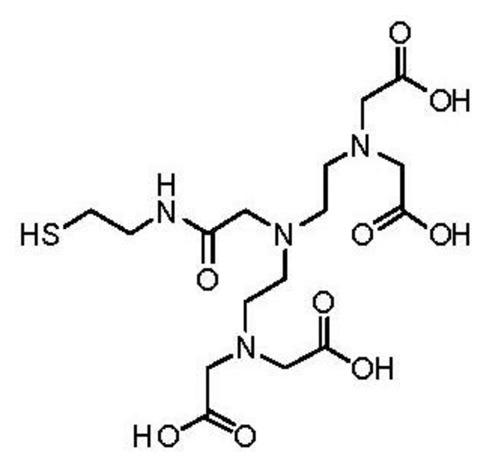


### 2.4. Preparation of Aβ_16–23′_ Solutions and Aβ_1–40_

Lyophilized Aβ_16–23′_ peptide was dissolved in acetic acid (about 10 μL) and Mili-Q water (30 μL). The concentration was determined using the BCA protein assay kit (Pierce, Rockford, IL, USA) which combines the well-known reduction of Cu^2+^ to Cu^1+^ by protein in an alkaline medium with the highly sensitive and selective colorimetric detection of the cuprous cation (Cu^1+^) by bicinchoninic acid [29].

Lyophilized Aβ_1–40_ peptide was dissolved in Mili-Q water (*ca.* 30 μL). The pH was immediately adjust to pH = 2 by adding 0.1 M HCl. The concentration of Aβ_1–40_ peptide was determined spectrophotometrically.

Next, the both peptides were diluted using TRIS buffer (50 mM TRIS hydrochloride, 300 mM NaCl) in order to get the stock solution concentration 200 and 133 μM, for Aβ_1–40_ and Aβ_16–23′_, respectively. The pH was adjusted to 7.4 by adding few μL of 0.1 M NaOH. Aβ_16–23′_ or Aβ_1–40_ solutions were aliquoted into sterile Eppendorf tubes and kept at −20 °C prior to use.

### 2.5. Preparation of Human Plasma

Natural human plasma obtained from the Regional Centre of Blood-Donation in Olsztyn was filtered with a Millipore Ultracel YM-3 and centrifuged for 60 min at 10,000 *g* rcf in order to remove proteins with molecular weight over 30 kDa. The filtered plasma was diluted twice with a TRIS buffer (50 mM TRIS hydrochloride, 300 mM NaCl, pH 7.4).

### 2.6. Preparation of Biosensor—Successive Steps of Gold Electrode Modification

Gold disk electrodes of 2 mm^2^ area [Bioanalytical Systems (BAS), West Lafayette, IN, USA] were used for the experiments. Gold electrodes were initially cleaned mechanically by polishing with alumina slurries (Alpha and Gamma Micropolish; Buehler, Lake Bluff, IL, USA) with particles sizes of 0.3 and 0.05 μm on a micro cloth pad (BAS) for 5 min each. Afterwards, they were carefully rinsed with Milli-Q water. The polished electrodes were further cleaned electrochemically by cyclic voltammetry. At first, they were dipped in 0.5 M KOH solution and swept with a potential between −400 mV and −1,200 mV against the Ag/AgCl reference electrode (with 3 M KCl inner solution) and the platinum wire counter electrode with a scan rate of 100 mVs^−1^, and the number of cycles: 3, 50 and 10, respectively. Next, the electrodes were cleaned in 0.5 M H_2_SO_4_, and CVs in the potential window between −300 mV and +1,500 mV were recorded, with the number of cycles: 3, 10 and 3, respectively, until the CVs were not changed. Before modification, the surfaces of electrodes were refreshed in 0.5 M KOH solution for 10 cycles. Clean gold electrodes were immersed in mixed solution of 10^−5^ M of pentetic acid–DPTA thiol ligand and 10^−3^ M of N-acetylcysteamine (NAC) in an ethanol/water mixture (80:20, v/v) at 4 °C for 20 h. Next, after washing with an ethanol/water mixture, water, methanol and chloroform-methanol solution (1:1), the electrodes were dipped in 1 mM copper (II) acetate solution in chloroform-methanol mixture (1:1) for 3 h. Then, after washing in chloroform-methanol solution, methanol and TRIS buffer 50 μM His_6_-RAGE V domain solution in TRIS buffer (10 μL) was dropped on the surface of the electrodes. The electrodes were covered and stored for 24 h at a temperature 4 °C. The buffer composition was as follows: 100 mM TRIS–HCl, 500 mM NaCl, pH 7.4 for His_6_–RAGE V natural domain and 100 mM TRIS–HCl, 300 mM NaCl, pH 7.4 for His_6_–RAGE V mutant domain. After deposition of the His_6_–RAGE V or His_6_–RAGE VC1 domain, the electrodes were washed and stored at 4 °C in TRIS buffer until used. Next, 10 μL of Aβ_16–23′_ solutions in TRIS buffer at concentration range: 0.001 μM, 0.010 μM, 0.100 μM, 1.000 μM were deposited onto the electrode surface for 30 min. After deposition of particular Aβ_16–23′_ or Aβ_1–40_ solutions, the electrodes were carefully washed and stored until use in 50 mM TRIS hydrochloride, 300 mM NaCl, pH 7.4).

### 2.7. Cyclic Voltammetry (CV) and Osteryoung Square Wave Voltammetry (OSWV) Measurements

All electrochemical measurements were performed with a potentiostat–galvanostat AutoLab (Eco Chemie, Utrecht, The Netherlands) with a conventional three electrode configuration. The following three-electrode configuration was applied: a gold electrode (BAS) as the working electrode, an Ag/AgCl reference electrode and a Pt counter electrode. OSWV was performed in a potential window from +0.7 V to −0.3 V and with a step potential of 0.001 V, a square-wave frequency of 25 Hz, and amplitude of 0.05 V. CV was performed in potentials from +0.7 V to −0.2 V with the scan rate of 0.1 V/s in the presence of 0.1 M KCl and 0.1 M KCl plus 1 mM K_3_[Fe(CN)_6_]/K_4_[Fe(CN)_6_]. The biosensor responses towards Aβ_16–23′_ or Aβ_1–40_ solutions, were expressed as the relative changes of Cu(II) redox currents: I_n_–I_0_)/I_0_ × 100%; where In is the Cu(II) redox peak current measured in the presence of the analyte and I_0_ the Cu(II) peak current measured in buffer without analyte.

### 2.8. Atomic Force Microscopy (AFM)

**Figure 1.**
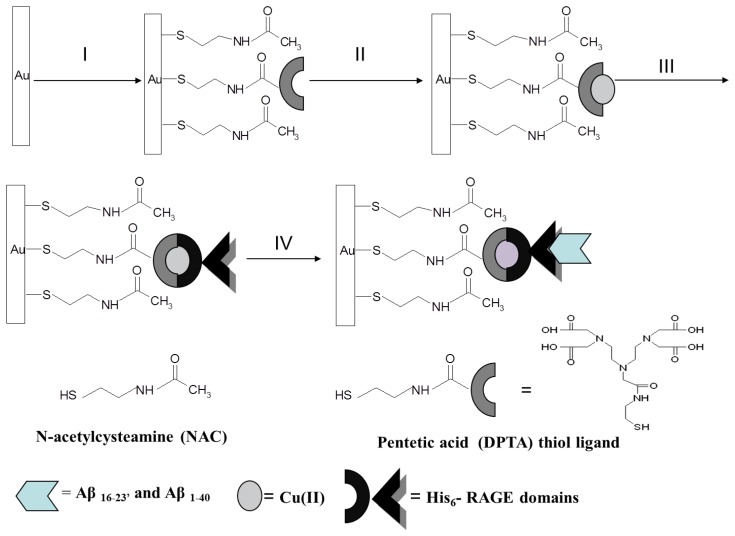
The scheme of the biosensor based on NAC/DPTA–Cu(II) SAM.

Each step of the gold substrates modification (see procedures described above and Figure 1) was characterized by an AFM system from Universal SPM Quesant (Agoura Hills, CA, USA).

Au(111) thin films on mica with gold thickness of 200 nm were used for modification (Phasis, Geneva, Switzerland). The AFM images were obtained using the intermittent – contact mode with NSC16 tip (W_2_C, Si_3_N_4_), which oscillates at *ca.* 170 kHz resonance frequency above the sample surface. The nominal spring constant of these cantilevers was equal 45 N/m. The radius of tip curvature was *ca.* 10 nm. The images were recorded in air with pixel resolution of 300 × 300.

## 3. Results and Discussion

### 3.1. Characterization of Electrochemical Biosensor Based on NAC/DPTA-Cu(II) Monolayer

**Figure 2.**
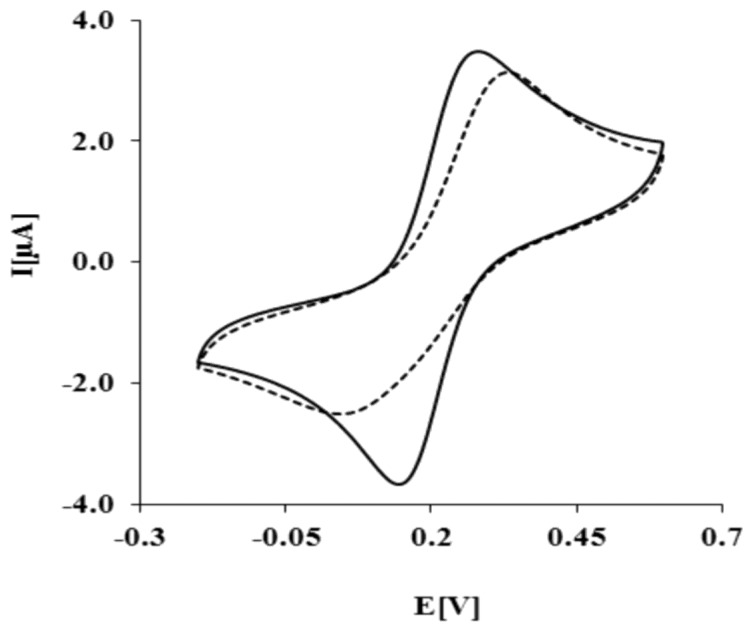
The CV curves measured for gold electrode: bare (solid line), or modified with NAC/DPTA–Cu(II) SAM (dashed line). Solution composition: 0.1 M KCl + 1 mM [Fe(CN)_6_]^3−/−4^. Measuring conditions: reference electrode Ag/AgCl, counter electrode Pt; scan rate 100 mVs^−1^.

The biosensor preparation scheme is presented in Figure 1. Each modification step was confirmed by electrochemical methods. As expected, K_3_[Fe(CN)_6_]/K_4_[Fe(CN)_6_] shows reversible behavior on a bare Au electrode, with a peak-to-peak separation ΔE_p_ = 79 ± 2 mV (Figure 2, solid curve). After the covalent attachment of NAC/DPTA SAM, reversibility of the system decreased with peak-to-peak separation ΔE_p_ equal to 158 ± 8 mV (Figure 2, dashed curve).

**Figure 3.**
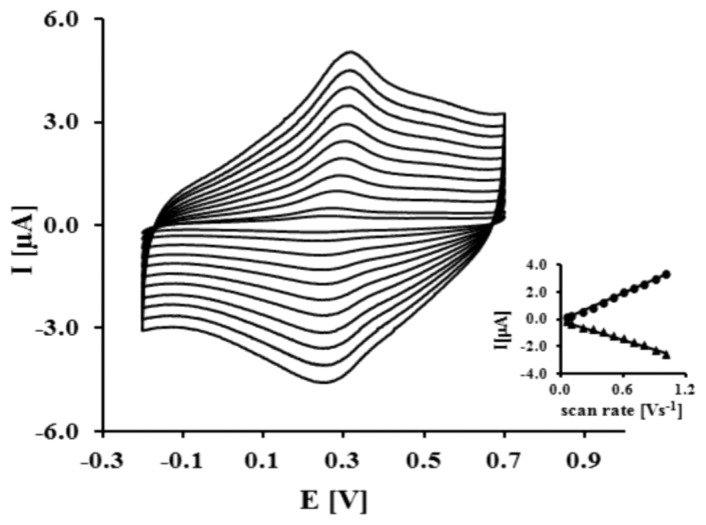
CV curves for gold electrodes modified by NAC/DPTA–Cu(II) SAM measured in 0.1M KCl. Scan rates: 50–1,000 mVs^−1^.

This result indicates that NAC/DPTA forms a well-packed monolayer on the surface of a gold electrode. DPTA was diluted with *N*-acetylcysteamine (NAC), which serves as a spacer molecule to avoid intramolecular interaction between the Cu(II) redox centers. The presence of Cu(II) at the surface of the electrode was checked using cyclic voltammetry performed in 0.1 M KCl. Representative CVs are presented in Figure 3.

The reduction and oxidation of Cu(II) were observed at E_pc_ = 262 ± 7 mV and E_pa_ = 320 ± 5 mV, respectively. The peaks separation ΔEp = 58 ± 10 mV indicates that the quasi-reversibility of the redox process of a DPTA–Cu (II) complex located at the electrode surface. The linear relationship of the anodic and cathodic peak currents versus scan rate from 50 up to 1,000 mVs^−1^ indicates that the redox process is not diffusion dependent and confirms the presence of the DPTA–Cu(II) complexes on the gold electrode surface.

The kinetic data of the electron transfer between the Cu(II) centers and the modified electrode surface were obtained from the relationship between the log scan rate versus the anodic and cathodic peak potential. The value of the electron transfer coefficient α = 0.34 ± 0.03 and the rate constant k = 0.91 ± 0.27 s^−1^ were calculated based on the generally applicable Laviron's procedure [30,31]. The Cu(II) surface coverage Г value we have found as 2.13 ± 0.9 × 10^−10^ mol cm^−2^. These data are in good agreement with parameters concerning dipyrromethene –Cu(II) and DPTA–Cu(II) SAMs already reported [32,33].

**Figure 4.**
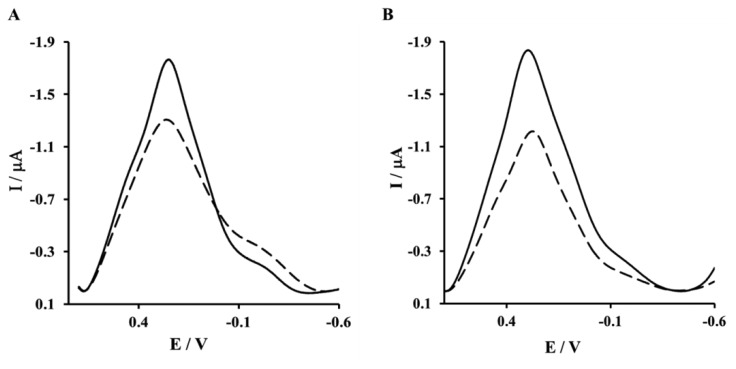
Square wave voltammograms of a gold electrode modified by NAC/ DPTA-Cu(II) (solid line) and NAC/DPTA-Cu(II)-His_6_-RAGE V domain (dashed line); (**A**) RAGE V natural domain; (**B**) RAGE V mutated domain. Measurement conditions: 0.1 M KCl, scan rate100 mVs^−1^.

The next step of sensor preparation was the immobilization of His_6_–RAGE V or His_6_–RAGE VC1 (natural or mutated) domains on the surface of a NAC/DPTA–Cu(II) monolayer. The immobilization was monitored by Osteryoung square-wave voltammetry. This technique was applied instead of cyclic voltammetry in order to reduce the capacitive current. The Cu(II) redox peak current was observed at 292 ± 8 mV. The complexation reaction between Cu(II) and His_6_–RAGE V natural or mutated domains caused the decrease of Cu(II) redox peak (Figure 4A,B). Similar results were observed for immobilization of His_6_–RAGE VC1 domain (results not shown).

### 3.2. AFM Characterization of NAC/DPTA-Cu(II)-His_6_-RAGE V Domain SAM

In order to visualize the topology of the gold surfaces after modification with SAMs according to the procedures described in the Experimental Section the intermittent contact mode of AFM in air was used.

**Figure 5.**
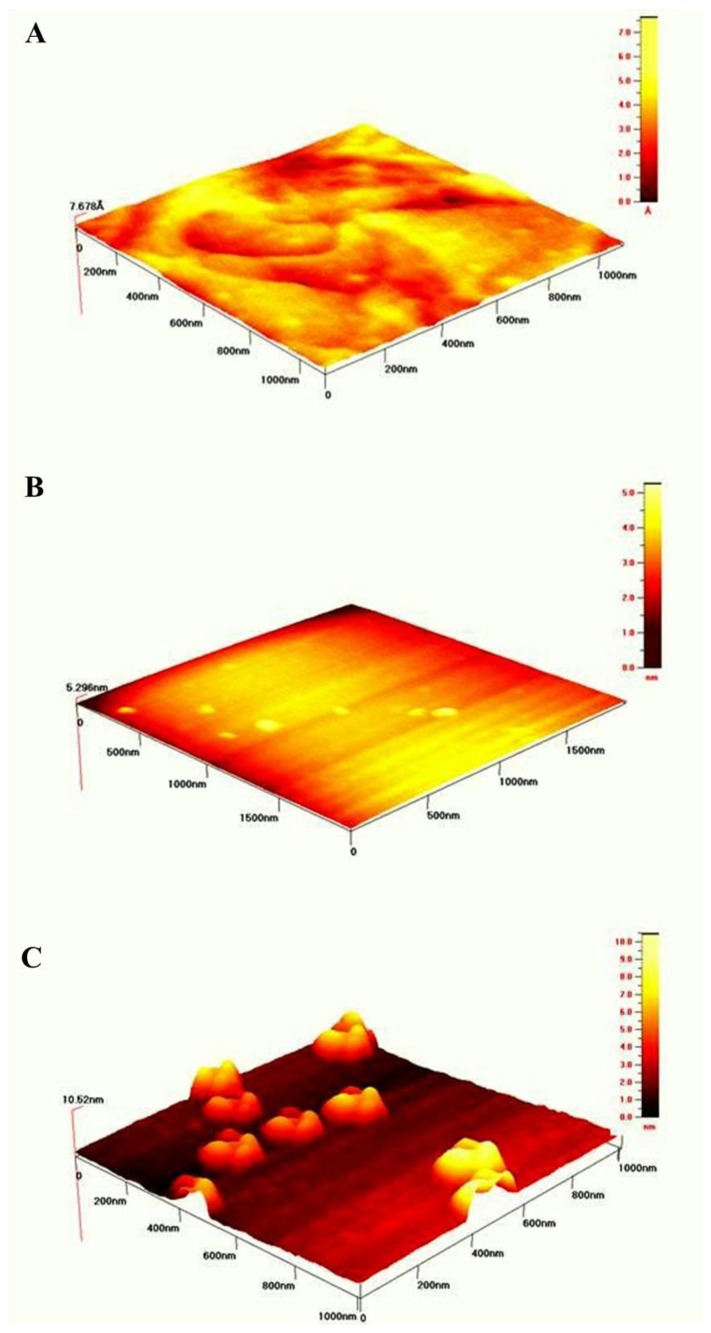
AFM images of: (**A**) gold Au(111) thin films on mica substrate, (**B**) NAC/DPTA SAM, (**C**) NAC/DPTA–Cu(II)–His_6_–RAGE V natural SAM.

Figure 5A illustrates the well formed terraces of bare gold surface. Its roughness was estimated over 1 μm^2^ area was equal to 3.3 Å The topography of gold surface modified with mixed NAC/DPTA SAM is pictured in Figure 5B. The value of roughness is equal 1.4 ± 0.2 nm. The topography of NAC/ DPTA–Cu(II)–His_6_–RAGE V natural domain SAM deposited on Au mica plate is illustrated in Figure 5C. Well separated objects with width: 131.4 ± 10.7 nm, length: 147.6 ± 11.2 nm and height: 5.9 ± 0.6 nm are visible. The amount of RAGE V domains relays on the amount of DPTA-Cu(II) complexes. We have found that the optimum molar ratio between DPTA and NAC was 1:100. A similar structure was observed for the His_6_–RAGE V natural domain immobilized on NAC/dipyrromethene–Cu(II) SAM [34].

### 3.3. Exploring the Interactions between V and VC1 RAGE Domains and Aβ Peptides (Aβ_16–23′_ and Aβ_1–40_) Using OSWV

**Figure 6.**
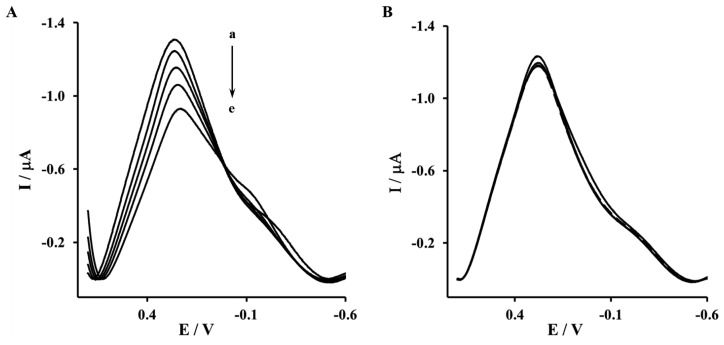
The OSWV responses of: (**A**) Au-NAC/DPTA–Cu(II)–His_6_–RAGE V natural domain, (**B**) Au-NAC/DPTA–Cu(II)–His_6_–RAGE V mutated domain towards Aβ_16–23′_ in the presence of 50 mM TRIS hydrochloride, 300 mM NaCl, pH 7.4. (a) 0.000, (b) 0.001, (c) 0.010, (d) 0.100, (e) 1.000 (μM).

**Figure 7.**
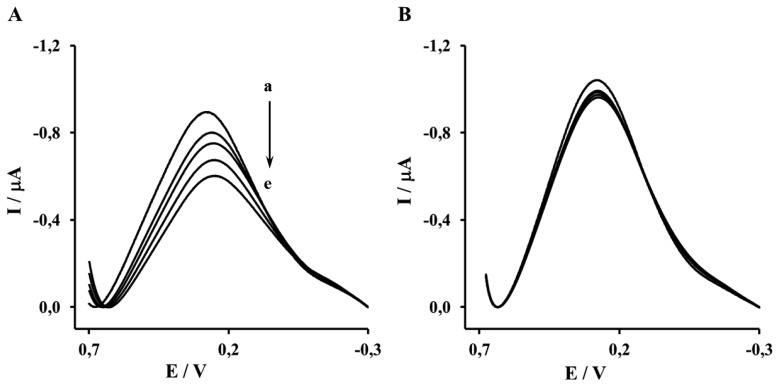
The OSWV responses of: (**A)** Au-NAC/DPTA–Cu(II)–His_6_–RAGE VC1 natural domain, (**B**) Au-NAC/DPTA–Cu(II)–His_6_–RAGE VC1 mutated domain towards Aβ_1–40_ in the presence of 50 mM TRIS hydrochloride, 300 mM NaCl, pH 7.4: (a) 0.000, (b) 0.001, (c) 0.010, (d) 0.100, (e) 1.000 [μM].

The Aβ_16–23′_ and Aβ_1–40′_ were determined in the concentration range from 0.001 to 1 μM. The representative OSWV recorded for gold electrodes modified NAC/DPTA-Cu(II)-His_6_-RAGE V domain SAM and NAC/DPTA-Cu(II)-His_6_-RAGE VC1 domain SAM are presented in Figures 6 and 7, respectively.

The highest concentration of Aβ_16–23′_ caused a 26.2% ± 4.6% decrease of the peak current recorded for the electrode incorporating the His_6_–RAGE V natural domain and 7.1% ± 2.8%, for electrode incorporating the His_6_–RAGE V mutated domain (Figure 6, Table 1). The potential at maximum Cu(II) redox peak current was slightly shifted into more negative direction. The sensitivity of the analytical system based on NAC-DPTA-Cu(II) used for covalent immobilization of His_6_-RAGE V domain towards Aβ_16–23′_ is quite good, but a little lower in comparison to the analytical approach based on NAC/dipyrromethene-Cu(II) [34,35].

The response of the electrode incorporating the RAGE VC1 domain towards Aβ_1-40_ was stronger in comparison to the response recorded for the electrode incorporating the RAGE V domain in the presence of Aβ_16–23′_ (Figure 7, Table 1). The highest concentration of Aβ_1-40_ caused 32.4% ± 4.1% and 8.4% ± 1.9%, decrease of peak current recorded for the electrode with His_6_–RAGE VC1 natural and mutated domains, respectively. The decrease of Cu(II) redox peak current in the case of His_6_–RAGE VC1 natural was assisted by a peak potential shift into the negative potential direction, whereas in the case of the His_6_–RAGE VC1 mutated domain, the peak potential remained almost unchanged in the presence of Aβ_1–40_ in the whole concentration range. This might indicate that application of two joined RAGE domains: the V-type and the trans-membrane C1 type is more suitable for biosensor performance.

**Figure 8.**
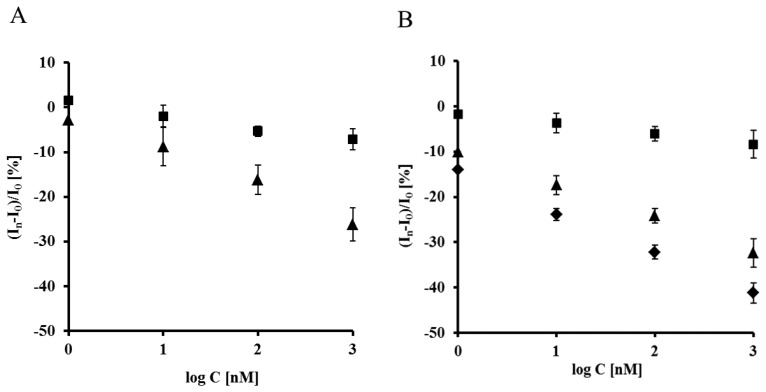
The relationship of relative Cu(II) redox current decrease *vs.* concentration of: (**A**) Aβ_16–23′_ measured with electrodes: Au/NAC/DPTA–Cu(II)–His_6_–RAGE V natural domain (▴)-in TRSI buffer; Au/NAC/DPTA–Cu(II)–His_6_–RAGE V mutated domain (▪)-in TRIS buffer (n = 7); (**B**) Aβ_1–40_ measured with electrodes: Au/NAC/DPTA–Cu(II)–His_6_–RAGE VC1 natural domain (▴)-in TRIS buffer, (♦)-in the presence of diluted human plasma; Au/NAC/DPTA–Cu(II)–His_6_–RAGE VC1 mutated domain (▪)-in TRIS buffer (n = 7).

Both electrodes displayed linear relationships between relative Cu(II) redox current decrease *vs.* the concentration of the peptides studied in the 0.001–1 μM range. They were also very selective. The electrodes incorporating mutated RAGE domains generated *ca.* three times smaller responses towards both analytes in the comparison to electrodes based on natural RAGE domains (Figure 8). The detection limits (DL) were calculated on the basis of equation (S/N = 3.0) [36]:

(1) DL=3.3×σ/S

where σ is the standard deviation of the response and S is the slope of the calibration curve. The obtained values of the analytes present in TRIS buffer were 1.47 nM and 1.06 nM for Aβ_16–23′_ and Aβ_1–40_, respectively.

In order to check the applicability of the biosensor proposed, the responses of different concentration of Aβ_1-40_ were determined in the presence of filtered plasma diluted twice with a TRIS buffer (50 mM TRIS hydrochloride, 300 mM NaCl, pH 7.4), using an electrode incorporating the RAGE VC1 domain. The components present in human plasma did not influence the biosensor performance. On the contrary, slightly higher responses were observed and the lowest detection limit (0.80 nM) was recorded (Figure 8B, Table 1). This value reaches the concentration level of Aβ_1–40_ which is relevant for determination of Aβ soluble form in human plasma [37], as well as in brain [37,38]. Therefore it might be concluded that the proposed electrochemical biosensors based on His_6_-RAGE V and VC1 domains covalently attached to the DPTA-Cu(II) complex deposited on gold electrode surface, are suitable for determination of Aβ_1–40_ in physiological samples.

### 3.4. The Mechanism of Electrochemical Signal Generation by a Biosensor Based on NAC/DPTA-Cu(II)-His_6_-RAGE Domain upon Binding Aβ Peptide

Electrochemical sensors based on redox active layers represent a relatively new direction in sensing device development. Their main advantage is the lack of the necessity of using external redox markers. The application of redox centers in a sensor gives very interesting possibilities. They can simultaneously act as host molecules and as well as transducers. It was already reported that the recognition reaction between a receptor immobilized on the redox active sensing layer and specific target molecules present in the sample solution might cause a change in the microenvironment surrounding the protein, which influences the double layer structure [39]. The model and potential distribution of the electroactive film was provided by Ohtani [40,41]. In the algorithm to compute the voltammetric responses of quasi-reversible system for a surface-tethered redox species, the double layer effect on the electron transfer kinetics and ion-pair formation between surface confined redox species and electrolyte ions were considered. The effects of the position of the redox centers immobilized onto electrode surfaces, as well as the effect of the ions present in the electrolyte on the thermodynamics of redox active monolayers were reported by Rowe and Creager [42,43]. They discovered that the distance of ferrocene units from the electrode surface as well as the presence of different anions in the basic electrolyte strongly influence the voltammetric response of redox active monolayer. The alternation of ferrocene electrochemistry caused by antibody binding to the sensing redox active interface was described by Gooding *et al.* [44]. The authors concluded the base of phenomenon observed was the restriction of counter ions accessing the redox sites to balance the charge.

A biosensor using the pentetic acid derivative DPTA was applied for creation of Cu(II) complex which was suitable for binding of His–tagged RAGE domains. We concluded that the base of working mechanism of the proposed biosensors is the change of RAGE domain conformation, which occurred upon binding of the Aβ peptides. This caused a restriction of counter ions accessing the redox probe to balance the charge, thereby hindering the electron transfer.

Similar phenomena were observed for the biosensors incorporating derivatives of iminodiacetic acid as well as dipyrromethene Cu(II) redox active SAM [33,34]. They have been applied for the stable and oriented immobilization of His–tagged receptor proteins. Upon stimulation with target molecules, a decrease of Cu(II) redox current was observed as the results of conformation changes of receptor proteins, which leads to the changes of electrolyte ions accessibility towards the redox centers.

## 4. Conclusions/Outlook

In this work we have presented electrochemical biosensors able to detect Aβ_16–23′_ and Aβ_1–40_ peptides in a concentration range between 0.001–1 μM. The surface of gold electrodes modified with NAC/DPTA–Cu(II) monolayer was suitable for oriented and stable immobilization of His_6_–RAGE V as well VC1 domains.

It was shown that the DPTA–Cu(II) complex located between the gold surface and the His_6_–RAGE V or His_6_–RAGE VC1 domains plays a double role: as an molecular connector for oriented and stable immobilization of His_6_–RAGE domains without changes of their biological activity, and as a transducer of the intermolecular recognition process between the RAGE V and RAGE VC1 domains and the Aβ_16–23′_ and Aβ_1–40_ peptides. The presence of human plasma did not influence the determination of Aβ_1–40_ which could be a useful marker for early diagnosis of Alzheimer's disease.

Taking into account the good analytical parameters such as sensitivity, selectivity, detection limit in nM range, and lack of responses towards human plasma components, the presented biosensor could be included to the measuring tools suitable for cost-effective theranostics.

## Figures and Tables

**Table 1.** The relative Cu(II) redox changes [%] current and peak potential shift [mV] measured with electrodes incorporated His_6_- RAGE V and VC1 domains after incubation with Aβ_16–23′_ and Aβ_1–40_ solution. (I_n_–current measured in the presence of particular peptide concentration, I_0_–current in the measured in buffer with no analyte; n = 7).

**C[μM]**	**Aβ_16__–__23__′_in TRIS Buffer**				**Aβ_1__–__40_ in TRIS Buffer**				**Aβ_1__–__40_ in the Presence of Diluted Human Plasma**	

	**His_6_-RAGE V natural**		**His_6_-RAGE V mutant**		**His_6_-RAGE VC1 natural**		**His_6_-RAGE VC1 mutant**		**His_6_-RAGE VC1 natural**	

	**(I_n_−I_0_)/I_0_** **[%]**	**Shift Potential** **[mV]**	**(I_n_−I_0_)/I_0_** **[%]**	**Shift Potential** **[mV]**	**(I_n_−I_0_)/I_0_** **[%]**	**Shift Potential** **[mV]**	**(I_n_−I_0_)/I_0_** **[%]**	**Shift Potential** **[mV]**	**(I_n_−I_0_)/I_0_[%]**	**Shift Potential[mV]**
0	0.0	266.0 ± 4.4	0.0	263.3 ± 9.0	0.0	280 ± 1.4	0.0	272.8 ± 2.5	0.0	259.0 ± 5.7
0.001	−2.9 ± 4.3	267.0 ± 4.4	1.6 ± 2.4	252.5 ± 5.4	−10.1 ± 2.1	265.5 ± 0.7	1.7 ± 1.7	273.8 ± 3.3	−14.0 ± 1.3	246.0 ± 5.6
0.01	−8.8 ± 3.3	264.0 ± 7.0	−2.0 ± 1.2	259.7 ± 8.5	−17.4 ±1.6	256.0 ± 8.2	−3.7 ± 0.7	270.5 ± 6.4	−23.9 ± 1.5	240.3 ± 5.6
0.1	−16.3 ± 3.7	249.5 ± 0.7	−5.4 ± 2.4	251.0 ± 9.4	−24.2 ± 3.1	249.0 ± 6.0	−6.1 ± 1.3	269.8 ± 5.7	−32.2 ± 2.2	227.8 ± 4.1
1	−26.2 ± 4.6	235 ± 5.7	−7.1 ± 2.8	244.5 ± 9.0	−32.4 ± 4.1	251.3 ± 4.7	−8.4 ± 1.9	268.3 ± 5.7	−41.2 ± 2.6	213.3 ± 3.4
